# Factors Facilitating Adoption of Pharmacogenetic Testing by Prescribers of Antidepressants in Four US Health Systems: A Multi-Site Cross-Sectional PGx Implementation Science Study

**DOI:** 10.3390/jpm16080411

**Published:** 2026-07-30

**Authors:** Alice B. Popejoy, Deborah Cragun, Megan C. Roberts, Lisa M. Bendz, Sarah Gonzales, Susanne B. Haga, R. Ryanne Wu, Natasha J. Petry, Laura B. Ramsey, Ryley Uber, Kaniz Momin, Nina R. Sperber

**Affiliations:** 1 Department of Public Health Sciences, UC Davis School of Medicine, University of California at Davis, Davis, CA 95616, USA; 2 Department of Community Health Sciences, College of Public Health, University of South Florida, Tampa, FL 33612, USA; dcragun@usf.edu; 3 Eshelman School of Pharmacy, University of North Carolina at Chapel Hill, Chapel Hill, NC 27599, USA; megan.roberts@unc.edu (M.C.R.); kaniz.momin@unc.edu (K.M.); 4 Center for Medication Policy and Drug Information, Department of Pharmacy, Duke University Hospital, Durham, NC 27710, USA; lisa.bendz@duke.edu; 5 Department of Population Health Sciences, School of Medicine, Duke University, Durham, NC 27710, USA; sarah.e.gonzales@duke.edu; 6 Center for Precision Health, School of Medicine, Duke University, Durham, NC 27710, USA; susanne.haga@duke.edu; 7 Department of Medicine, Division of General Internal Medicine, School of Medicine, Duke University, Durham, NC 27710, USA; ryanne.wu@duke.edu; 8 23andMe, Palo Alto, CA 94306, USA; 9 Sanford Health Imagenetics, Sioux Falls, SD 57105, USA; natasha.petry@sanfordhealth.org; 10 Department of Pharmacy Practice, North Dakota State University, Fargo, ND 58108, USA; 11 Division of Clinical Pharmacology, Toxicology and Therapeutic Innovation, Children’s Mercy Hospital, Kansas City, MO 64108, USA; lramsey@cmh.edu; 12 Center for Pharmacy Innovation and Outcomes, Geisinger, Danville, PA 17822, USA; ruber@geisinger.edu; 13 Center of Innovation to Accelerate Discovery and Practice Transformation (ADAPT), Durham VA Health Care System, Durham, NC 27705, USA

**Keywords:** adoption, antidepressants, coincidence analysis (CNA), diffusion of innovation, genetic testing, implementation science, pharmacogenetics, psychiatry, serotonin and norepinephrine reuptake inhibitors

## Abstract

**Background**: Pharmacogenetic (PGx) testing could identify actionable drug–gene interactions, reducing the risks of inappropriate prescribing of certain medications in some patients. An area of growing public health concern is rising global rates of depression and antidepressant use over the last two decades. Prior research has elucidated perspectives of healthcare providers who prescribe antidepressants regarding the clinical utility of genetic information, including PGx testing, but there is a gap in understanding how individual perspectives and systemic contextual factors may combine to influence PGx testing adoption. **Objective**: The objective of this study was to elucidate combinations of individual and contextual conditions associated with willingness to adopt PGx testing for Cytochrome P450 Subfamily IID, Polypeptide 6 (*CYP2D6*) and Subfamily IIC, Polypeptide 19 (*CYP2C19*) among antidepressant prescribers. **Methods**: We conducted a cross-sectional, mixed-methods study using structured questionnaires and semi-structured interviews with healthcare providers who prescribe antidepressants within their scope of practice across four healthcare systems in the United States. We collected data on implementation science concepts from the Theoretical Domains Framework, the Consolidated Framework for Implementation Research (CFIR), and the Implementation Outcomes Framework. Coincidence analysis (CNA), a case-based, Boolean logic-based method that identifies minimally sufficient combinations of conditions that lead to a particular outcome, was used to identify combinations of conditions for PGx test adoption among antidepressant prescribers. Interviews were also conducted with 10 patients who received pharmacogenetic testing within these healthcare systems to contextualize findings with patient perspectives. **Results**: Prescribers adopted PGx testing when they believed it would be beneficial to patients and were not deterred by cost-related concerns; the combination of these conditions led to PGx adoption in the most highly supported CNA model. Patient perspectives were also consistent with the selected model, with data suggesting they may have greater willingness to tolerate costs when they perceived or experienced benefits from testing. **Conclusions**: Insights from this study may be used by health system administrators and public health policymakers to inform future PGx implementation strategies that enhance uptake and awareness of existing evidence for clinical benefits of PGx testing and mitigate cost-related barriers to adoption.

## 1. Introduction

*Pharmacogene* variants—genes that are involved in drug metabolism, transport, absorption, and/or excretion—influence variability in how patients respond to medications such as antidepressants, which represents a growing public health issue as increasing numbers of individuals are prescribed these drugs in the United States and globally [1,2,3]. Research suggests that PGx testing interventions conducted through primary care could help prescribers better meet patients’ needs by improving the process of identifying, tailoring, and adjusting treatment plans for depression and other mental health conditions [4]. Studies on patient perspectives also suggest that many find PGx testing helpful and valuable for their healthcare, while also highlighting potential risks and concerns [5,6,7,8].

Pharmacogenetic (PGx) testing can inform the process of prescribing certain drugs with known genetic interactions, especially among patients who may have a higher risk of adverse events, for example, patients whose genetic backgrounds are underrepresented among participants in phase I and II clinical trials demonstrating drug safety and efficacy [9,10,11]. One multi-site study of ~7000 patients seen in hospitals, community health centers, and community pharmacies across seven European countries demonstrated a significant reduction in adverse drug reactions (ADRs) with the intervention of preemptive testing for known clinically actionable PGx variants using a 12-gene panel [12]. Given the abundance of these genes’ involvement in the pharmacokinetics (PK) of many drugs on the market, this strategic selection enhances their relevance to other clinical domains. In psychiatry, clinical PGx testing implemented to prevent ADRs to psychiatric medication has been shown to be cost-effective, reduce overall deaths, and improve patient quality of life [13].

Despite the increasingly robust evidence base for the demonstrated benefits of targeted PGx testing in psychiatry and other clinical domains for specific drug combinations, conditions, and patient genomic backgrounds [14,15,16,17,18], its adoption in routine clinical care has been slow or nonexistent, depending on the health system, prescriber, and context [19,20]. As such, additional research is needed to elucidate patient and prescriber perspectives on PGx testing, in combination with systemic or contextual factors that may interact with individual views and attitudes, to reveal the specific combination(s) of factors that contribute to the likelihood that clinical providers will adopt PGx testing in practice.

The overall objective of this study is to elucidate such combinations of factors using coincidence analysis (CNA) to generate models showing plausible pathways to the adoption of PGx testing for two cytochrome P450 genes (*CYP2D6* [MIM: 124030] and *CYP2C19* [MIM: 124020]) involved in the PK of commonly prescribed antidepressants [21]. Table 1 lists drugs with FDA-approved labels annotated by ClinPGx to contain clinically actionable PGx information on *CYP2D6* or *2C19*, and the respective regulatory agencies in Europe, Canada, or Switzerland that have approved drug labels consistent with those in the US. Restricting our focus to actionable gene–drug pairs helps isolate implementation issues related to the use of this information, rather than conflating adoption with uncertainty surrounding less established PGx applications in other therapeutic areas.

Prescriber-specific perspectives can influence the likelihood that PGx testing will be adopted [22,23] in practice, which suggests that interventions should be tailored to specific factors that are found to influence prescriber decisions to order PGx tests. Furthermore, contextual factors that are inconsistent across health systems and clinical domains, such as payer coverage and clinical guidelines for PGx testing, lead to variability in adoption trends [24]. Our study design and analytic approach enabled us to explore combinations of both individual-level factors and contextual conditions that must be considered together when evaluating tailored clinical pharmacogenetic implementation efforts.

## 2. Materials and Methods

Data for this cross-sectional, mixed-methods case study came from structured questionnaires and semi-structured interviews with 29 prescribers of antidepressants from four different healthcare systems in the United States (May–December 2022) and interviews with a convenience sample of 10 patients who received PGx testing within these healthcare systems and were available for interviews (August 2023–2024). The four participating health systems were purposively selected based on their participation in NHGRI-supported genomic medicine implementation networks (IGNITE and/or eMERGE) and to ensure the inclusion of providers who have and have not adopted PGx testing in a variety of different implementation settings, as previously described in Sperber et al. [25].

We focused on antidepressant prescribing because CPIC guidance was available for *CYP2D6*- and *CYP2C19*-related antidepressant prescribing decisions, and antidepressants are commonly prescribed in both primary care and psychiatry, allowing for the evaluation of PGx adoption across diverse real-world clinical settings. The distribution of providers by specialty is demonstrated in Table 2, with 69% (20/29) practicing mostly in primary care, 24.1% (7/29) in psychiatry, and 6.9% (2/29) in mixed internal medicine/psychiatry roles. All but two providers worked primarily in outpatient settings; these two worked in an inpatient setting.

The data resulting from prescriber responses were calibrated, or coded, to enable systematic evaluation using coincidence analysis (CNA) [26]. CNA is a case-based, Boolean logic-based configurational method used to systematically identify redundancy-free combinations of conditions that are sufficient for an outcome [27]. CNA can return a null result if no combination of minimally necessary and sufficient factors is identified.

We used qualitative data from prescriber interviews to calibrate factors for use in CNA results, drawing on a previously published qualitative analysis of the prescriber interview data [4]. The structured prescriber questionnaire was intended to supplement qualitative interviews and support case-level calibration for CNA, while interviews provided clarification and deeper exploration of survey responses. Patient participants included a convenience sample from two clinical sites in our study with opposite levels of readiness and support for system-wide PGx implementation. All interviews were conducted and recorded with Microsoft Teams.

Detailed descriptions of qualitative interviews with medication prescribers were provided in a prior publication [4] and served as the source material for factor value coding and model pathway analyses using CNA in this study. Analysis of the interview data revealed substantial repetition of themes across interviews, with few novel concepts emerging in later interviews. Analysts used NVivo14 v14.23.3 qualitative data analysis software and a priori implementation science concepts from the Theoretical Domains Framework [28], the Consolidated Framework for Implementation Research (CFIR) [29,30], and the Implementation Outcomes Framework [31] to code interview data for factors that may potentially influence adoption [32].

To review the coded data, we used the framework matrix tool in NVivo in which the rows consisted of cases (i.e., prescribers), the columns consisted of factors, and the cells summarized coded quotes about each factor at the case level. We also accessed full case transcripts within NVivo for more in-depth queries of qualitative data. To prepare the qualitative data matrix for use in CNA, two analysts worked together to assign numerical values representing qualitative differences in the factors for each case (a process known as calibration), which are defined in Table 3.

Calibration occurred in two phases that each involved discussions among two or three team members. In the first phase, values were encoded for neutral statements that were neither clearly positive nor negative; in the second phase, these were consolidated into fewer factor values, such that one numerical value represented the presence of each factor. For example, the concept coded as “benefits” was defined as prescribers’ beliefs about positive consequences and relative advantages of PGx; this was calibrated in the initial phase as “1” for negative statements only, “2” for mixed positive and negative statements, and “3” for positive statements only. In the final calibration, this was recoded as “0” for no benefits perceived and “1” for at least some perceived benefits.

This two-phase calibration process enabled nuanced conceptual considerations that were refined over time and reduced both data imbalances that occur when there are too few cases with each factor value and data fragmentation that occurs when multiple possible configurations are not represented in the data. Data fragmentation was further reduced by excluding two factors (i.e., provider knowledge and primary care setting), which were the least reliable data (knowledge) and relevant for implementation science (primary care), respectively. This left eight factors that were included in our final analysis, in addition to the outcome. PGx testing adoption (the outcome of interest) was calibrated as “1” if prescribers reported taking at least one of the three following actions within the past six months of their practice: (1) offering to order PGx test(s) for a patient; (2) using the results of a genetic test automatically generated by their clinical institution; or (3) referring patient(s) to a specialist to order a PGx test. A six-month timeframe was selected to balance opportunities for PGx use with recall accuracy. A shorter timeframe could misclassify clinicians who had adopted PGx testing but had not recently encountered an appropriate clinical scenario, whereas a longer timeframe could increase recall bias and capture practices that no longer reflected current use. All other prescribers interviewed were assigned a “0” to indicate non-adoption as the implementation outcome.

CNA models were generated using the cna R package (version 4.0.3) [26], with the fully calibrated case matrix (Table 4). The frscore [33] package (version 0.4.1) in R was used to automatically generate and compare CNA models across various parameter thresholds. Our final CNA used antecedent-adjusted consistency and antecedent-adjusted coverage to account for imbalances in the data, thresholds of fit.range = c (0.95, 0.75) and granularity = 0.05, with a maximum number of models set to 400 to ensure all models were used by frscore to calculate fit-robustness. Frscore uses nested (hierarchical) relationships among models to compute quantitative fit-robustness scores, thereby aiding the identification of robust candidate models, which help with model selection when multiple models fit the data (i.e., model ambiguity), which is common with CNA [34].

Throughout the model review and selection process, analysts queried the qualitative dataset to help interpret the models and ensure they were substantiated by the underlying observations. Due to the largely exploratory and qualitative nature of our approach, qualitative data analysts with deep knowledge and familiarity with provider interviews selected their preferred model using quotes to help elucidate the most plausible model.

When selecting among the most robust models, the CNA analysts prioritized low complexity and balanced performance across multiple measures of model fit rather than selecting the most robust model. Specifically, we reviewed several fit measures that range from 0 (low fit) to 1 (perfect fit). These fit measures include consistency (i.e., the degree to which a certain combination of causal conditions reliably predicts the presence of a specific outcome) and coverage (i.e., how well a given solution accounts for cases with the outcome), as well as variants of consistency and coverage that account for imbalances in the prevalence of the conditions. These consistency and coverage measures were treated as the primary fit measures with other fit measures used alongside consistency, coverage, complexity, and relative fit-robustness to compare and select among the models.

We also conducted qualitative analysis of interviews with patients to assess whether their responses would provide additional support for the selected model. Patient participants were eligible if they had been prescribed antidepressant medication and had received PGx testing. The patient interview guide was designed to elicit a broad array of information, including whether and how cost factored into their decisions to have testing and their views about perceived benefits of PGx testing. Data were analyzed using directed content analysis, in which data were coded by concepts queried via the interview guide [35]. The coded content was then reviewed to identify themes and evaluate convergence with the prescriber data.

## 3. Results

### 3.1. CNA Model Selection

The results of running CNA with frscore on our data produced 378 scored model types. The top ten most robust models are included in Table 5. Model 7 was selected by the qualitative analysts based on their knowledge of the interviews. This model was also the most well supported when considering all measures of model fit, combining strong consistency and coverage, superior contrapositive performance, and comparatively high faithfulness with low complexity.

**Figure 1:**
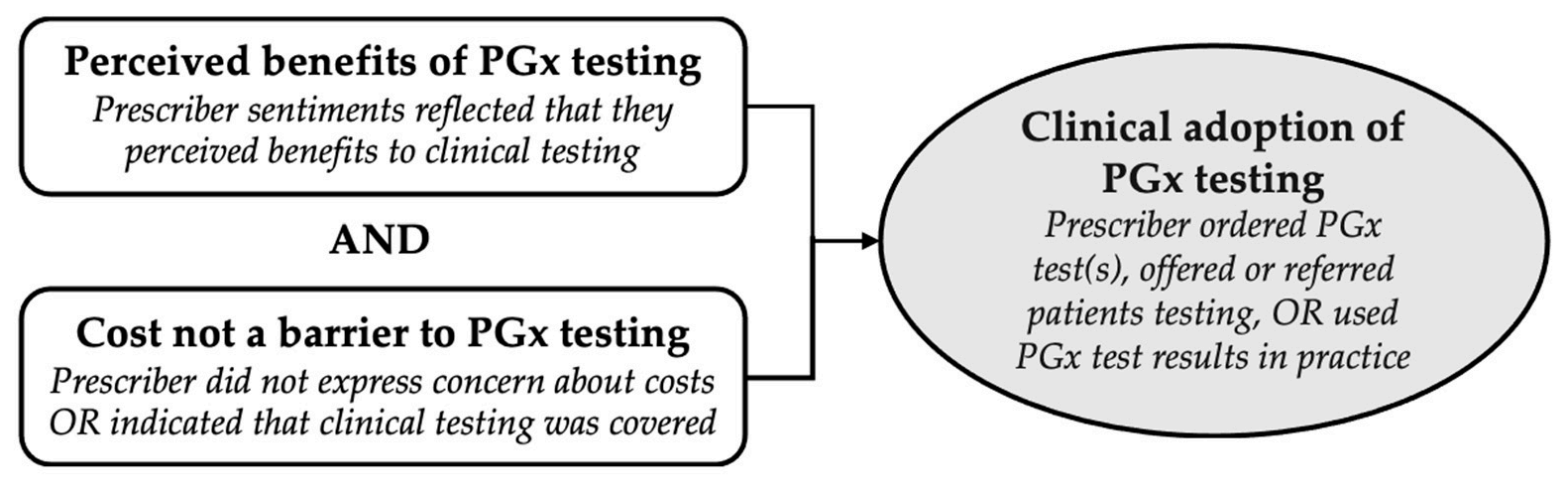
Visual depiction of factor values supported by CNA and qualitative evidence as necessary and sufficient for adoption of PGx testing for prescribing antidepressant medications. The combination of perceived benefits and cost not a barrier to testing lead to clinical adoption of PGx testing in this selected model.

Perceived benefits of PGx testing, combined with a lack of concern about costs, did a better job of distinguishing between adopters and non-adopters than the model with cost concerns alone. This model had the highest standardized consistency score (0.929) among the top ten most robust models and a standardized coverage of 1.0 as well as high scores on other models of fit (Table 5). Based on our selected model (Table 5; highlighted in gray), the presence of perceived benefits of testing almost always led to adoption if costs did not pose a barrier (Figure 1). This model indicates that perceived benefits of PGx testing together with cost not being a barrier were minimally sufficient (when combined) for antidepressant prescribers to adopt PGx testing in practice.

### 3.2. Qualitative Evidence Supporting Model Selection

Among the 29 clinical prescribers interviewed, 23 (79%) perceived benefits of PGx testing, including all 16 adopters (55%) and seven non-adopters (24%). These prescribers considered PGx testing to be more advantageous than the trial-and-error standard of care, indicating the following benefits:

- Less suffering for patients when it takes less time to achieve a therapeutic benefit;
- More objective information to select best-fit medications for individuals;
- Positive consequences consisted of engaging patients with medication selection;
- Improved access to mental health medications through primary care.

All 16 adopters indicated that out-of-pocket cost did not present a barrier for them to offer the test to their patients. Thirteen of the 16 PGx adopters (81%) said that they offer testing to patients regardless of the cost; patients then decide whether they would want to pay for it, if not already covered.

The other three adopters (19%) expressed concerns about costs of PGx testing in general yet opted for the test(s), explaining that their health system or an active clinical research project would cover the costs—so cost-related concerns were alleviated. However, they explained that cost would affect their decision to offer PGx testing to patients if it were not covered. In one of these cases, an outpatient primary care prescriber in a health system that did not have institutional support had adopted PGx testing during a study on the clinical effectiveness of PGx testing when it was offered at no cost to patients. When asked whether they would consider cost in their practice without the funded program, they said, “I would… if it weren’t covered by insurance, lab tests can be expensive…If it’s not covered by insurance, my patients probably aren’t going to get it done.”

The other two adopters who may have seen cost as a barrier in the absence of institutional support (such as health system subsidies for testing) were also outpatient primary care prescribers, with one noting the following:

“Yes, cost is always an issue…When [the health system] stopped offering [a health system covered test], I wasn’t sure if I ordered a panel if the person’s insurance will pay for it or if they will pay out-of-pocket. I actually partnered with a pharmacist to figure out if Medicare would pay for those tests…they were just going to submit it to Medicare and see if Medicare would cover it, but if they don’t the system was going to write it off for them…I would want to make sure it’s being covered by insurance and the patient will not have to pay at least a substantial amount out-of-pocket”.

This example showcases the lengths one prescriber would be willing to go to offer testing for a patient, presumably because they perceive it as beneficial, while ensuring that the costs are not a burden to their patient. The other prescriber from this same health system with subsidized PGx testing indicated that while they viewed pharmacogenetic testing as beneficial, they had concerns about discussing cost with patients and the overall impact of these costs in the absence of subsidies. Specifically, they expressed concern that a new patient might not return if they were to receive “a big bill” after their first visit, explaining that their clinical practice consists of “up front” communication with patients about how prescribing involves trial and error.

In contrast to adopters, non-adopters expressed divergent views about the benefits of testing. None of the 13 non-adopters indicated that they would offer it to patients, regardless of cost concerns. Six of the 13 non-adopters (46%) saw no benefits and also shared concerns about cost. For example, one non-adopter said, “It’s not going to give us information on how it’s going to make the patient feel,” and “I have not offered it to patients because of the cost.” Non-adopters also expressed doubts about whether testing would improve patients’ experiences or outcomes and shared concerns that it could potentially lead to misunderstanding by patients. For example, one said, “I feel like the results that I’ve seen are kind of nebulous, and it –sort of tells me, ‘Oh, this person’s a little bit higher metabolizer; this is a slower metabolizer.’ And then, I’m still going to start with a few medicines that I know and have a lot of clinical experience with…I don’t, I mean, really appreciate how that is helpful…”. Another prescriber (coded as a non-adopter of PGx testing) said, “I think that the misunderstanding is greater than the potential for benefit and so I just don’t bother.”

In contrast, six other non-adopters, all primary care, explicitly expressed concerns about potential out-of-pocket costs to patients and/or lack of reimbursement to the health system associated with testing, which prevented them from considering it further—even though they believed PGx testing might be beneficial. One of these prescribers said that while they did not offer PGx testing to their patients as part of their regular prescribing practice, they would use it when subsidized by patients’ health plans. They said, “I don’t think [we should do this] at this point because of [the lack of] coverage across the board.”

The remaining non-adopter viewed testing as beneficial in general but not for their pediatric population: “I’ve found that it has some limitations for us in pediatrics because kids only have indications for certain medications, and oftentimes with the tests that we’ve used in the past, sometimes, the preferred medication is not one that has indications for children. So, it really has been somewhat limited in terms of how helpful it’s been in pediatrics so far.”

### 3.3. Patient Perspectives on Costs and Benefits

We interviewed 10 patients in total. Five patients had received genetic testing from the health system that had the lowest amount of institutional support and five had received it from the health system that had the highest (see Table 2 for the distribution of participants by site). Because the patient interviews were conducted to provide contextual insight into the relevance of the selected CNA model to patient experiences with PGx testing, recruitment ceased after 10 interviews when sufficient information had been obtained for this purpose.

Patients’ willingness to pay an out-of-pocket cost for PGx testing depended in large part on the extent to which they could anticipate receiving a personally beneficial result. All ten patients described similar specific benefits as those expressed by prescribers of antidepressants. Specifically, five of the patients reported benefits like reducing “suffering”. Four of the patients described how genetic testing offered reliable information, which is grounded in scientific data. One patient said that they appreciated the interaction with their clinician about the results, specifically the time spent engaging with them to understand the implications for their medication management.

Two of the patients who regarded the results as directly impactful to them said that they would pay for the test. One described their perception of value:

“…my mom had a number of problems with arthritis, pain medications, with high blood pressure, and with statins. And it just seemed like a lot of times with the high blood pressure meds., they put her on one med. It wouldn’t work, so then they’d keep her on that med. and add another med. And it just seemed such a trial and error. And it was so frustrating to me because then you were kind of worrying is it some side effects from other ones happening when her potassium was going low. And just all these different things.

And so, I had made a vow to myself that before I started a med, I wanted to find out if it was good for my body or not. And is there a way to do that? Well, because I was in my parent’s … chart because I was their liaison and I had permission to go in their chart, I saw that you were offering … genetic testing. And the price that they had on there—I had talked to another friend who had some genetic testing that was very expensive through California, so I was very excited… I was so happy to hear it was $40. To me, that was nothing to find out this information.”

The remaining eight patients suggested that their willingness to pay would depend on perceived need for the test, for example, if they knew that the information was necessary to see an improvement in their health. One patient who lived on a fixed income and did not have any changes to their medications based on results said, “I mean if it was something without the testing that was going to interfere with my health, or there was something like that, then yes I would pay whatever it would cost—well, not whatever, but within means what it would cost me out of pocket to have it done.”

Another described how they regarded testing as beneficial for patients in general: “If the results were being used and done correctly, I really think that it could help a lot of people to know if they’re on the right med, or if they shouldn’t be on it, or if they should be on something else. I really think it could be beneficial.” On the other hand, this same patient did not experience a value for themselves: “[I] thought it was very interesting, but overall, it didn’t really change anything. I’m still on the same med. I’m still doing the same thing. So, as far as that goes, it didn’t make a difference for that. So, it being interesting, yeah, possibly worth it. But not really making any changes to my meds or anything, I don’t know if I would go as far as paying for it.”

It is notable that while this patient understood that the test would provide more information about the fit of their medication, they did not have a discussion with their ordering physician after receiving the results and described lacking interpretation. As they said, “I know he read it because he was like, ‘Oh, I see your test got back.’ And that was the end of it. And I probably should have said, ‘Well, I read this, this, this.’ But I just kind of wish it [the report] would have been like, ‘It says this, but we still think you’re on the right medication. We’ll just monitor and make sure…’”. This anecdote illustrates how patients may judge the value of a clinical PGx test according to the likelihood that it will impact their treatment and outcomes.

## 4. Discussion

Overall, our results underscore the need for system-wide strategies to address (1) prescribers’ willingness to discuss out-of-pocket costs with patients when not covered by an institution or health plan, (2) prescribers’ perceived benefits of PGx testing, and (3) patient education about the benefits (and limitations) of testing, as results do not always change the care they are provided. For a subset of clinical prescribers, future implementation strategies to motivate adoption of PGx testing may need to focus on changing beliefs about the benefits of testing or building skills for discussing costs with patients.

Given that the US Food and Drug Administration (FDA) has approved drug labels that contain clinically actionable pharmacogenetic information for certain drugs, there should be a concerted effort to increase the use of this information in practice [36]. ClinPGx [37] aggregates and publishes information on regulatory agency-approved drug labels that contain clinically actionable pharmacogenetic information. Among these, there are FDA-approved drug labels related to *CYP2D6* and/or *CYP2C19* for eight different antidepressants (Table 1).

Whereas many other studies have focused on the need to address cost concerns or enhance education and training of physicians to bolster prescriber use of PGx testing [38,39,40,41,42], our results suggest that both prescribers’ willingness to discuss testing costs with patients and their professional assessment of the benefits of testing to improve medication management and patient outcomes were needed for adoption of PGx testing. From these findings, we can infer that physician education about the benefits of PGx testing and training alone may not suffice to enhance adoption of PGx testing without tandem efforts to address prescribers’ concerns about costs to patients and health systems.

Previous findings suggest that prescriber willingness and comfort in having a conversation with patients about PGx testing may support adoption [4]. Notably, prescriber interviews revealed that some are concerned about the broader impacts and potential burden of testing costs on patients, including the strength and continuity of their patient–provider relationship. We found that some prescribers were concerned about a patient not returning to their clinical practice if faced with a large medical bill on the first visit. This provides further support for the need to consider the broader context of healthcare systems and the fiscal environments in which they operate when investigating and mitigating barriers to PGx implementation. Patient perspectives corroborated the importance of considering costs and benefits of testing, consistent with factors that appeared to make the difference for prescribers who adopted it in practice.

Given that about 95% of patients are likely to have some genetic variant that could expose them to a higher risk of an adverse drug event or the potential for lower efficacy of certain drugs (if prescribed) than the general population, it is critical to understand how we might increase adoption of PGx testing beyond the context of clinical psychiatry. While we collected data from prescribers who work in health systems with different levels of support for PGx testing, it is also important to understand how the broader fiscal and reimbursement infrastructure and socio-political environment in which healthcare systems are implemented might impact adoption. Some studies have identified cost as a barrier in healthcare settings with public payer systems, as in Quebec [43], Switzerland [44], and Australia [45]. However, most research on barriers to implementation of pharmacogenetic testing in countries with strong national healthcare systems do not cite individual patient costs as a major barrier [41,42,43,44,45,46,47,48].

One study [47] piloted a strategy to determine whether PGx tests would be covered by partnering with pharmacists, which could also be a potential pathway to adoption, either in partnership with prescribing physicians, or as an alternative approach to delivering clinically actionable information directly to patients, who could use this to self-advocate for more precision-oriented medication evaluation and increase prescriber awareness of its utility.

Our results suggest that prescribers would be more likely to adopt PGx testing in clinical or institutional environments where the practice is broadly supported, through coverage of tests by the health system or translational research grants, or through the culture and practices of a genomics-enabled learning health system (gLHS) [49].

If healthcare system administrators, leaders in academic medical centers, and local clinical prescribers had assurances that PGx tests would be covered by insurance companies, this would establish a solid foundation for future educational and implementation efforts, as cost considerations would no longer be a hindrance to adoption. One interesting case will be the state of California, in which the state legislature formally codified pharmacogenetic testing as a Medi-Cal benefit, effective 1 July 2024 [50]. It is yet to be determined whether and, if so, to what extent such public health insurance coverage policies influence clinical practice, trends in reimbursement, and patient outcomes over time.

### 4.1. Study Limitations

Our cross-sectional study design provided a point-in-time assessment and could not capture how adoption processes evolved over time. The study included prescribers from four purposively selected health systems, which may limit transferability to other settings. Although institutional support was included as a factor in the analysis, other unmeasured differences across sites and implementation contexts may have influenced adoption. Many factors were based on self-reported perceptions and experiences and may be subject to recall or social desirability bias. In addition, adoption was determined from prescribers’ self-reported behaviors because prescribing or order data were not available for independent verification.

Despite our confidence, based on qualitative data, that the selected CNA model represents the most salient pathway to adoption, there was substantial model ambiguity. Several alternative models demonstrated comparable fit, suggesting that additional factors may influence adoption.

The classification of adoption was not straightforward. Prescribers at one site that had a centrally funded initiative to subsidize PGx testing were more likely to use test results that were accessible in the patients’ records. It may be the case that prescribers in health systems or clinical settings with subsidized PGx testing programs would no longer be considered PGx testing adopters if they had been surveyed or interviewed after these programs ended, or in another system.

Because the study was designed to generate explanatory insights rather than representative estimates, both the provider and patient samples should be interpreted within the context of their intended analytic purposes. Our results are intended to provide insights relevant to implementation strategies for antidepressant prescribers, not to report representative estimates of provider attitudes within participating health systems. Additionally, the sample size for prescriber surveys and interviews is small relative to the size of the health systems in which they are employed, and our results should not be interpreted as asserting claims about system-level trends. Patient data were used to provide contextual insight into patient experiences with PGx testing across healthcare systems with differing levels of support for PGx implementation. Because patient interviews were not conducted at all study sites, they could not be used to corroborate provider findings within individual study sites. Furthermore, patient interviews were conducted with individuals who had already received PGx testing, and their perspectives may therefore differ from those of patients who declined testing or have no prior experience with PGx testing. Finally, patient perspectives were not linked to specific prescribers or prescribing decisions, limiting our ability to evaluate the relationship between patient experiences and prescriber adoption behaviors.

This study was situated in the United States, which does not have a single-payer healthcare system, and where individual patients are often responsible for covering the costs of expensive medical treatment and tests not covered by private insurance companies. To address this limitation, we recruited prescribers of antidepressants who had adopted genetic testing from clinical and healthcare settings with a range of institutional support for the tests. This introduced variability in the system-wide, institutional conditions surrounding PGx testing, including how prescribers perceived the burden of costs and/or likelihood of reimbursement. We accounted for this variability by including “institutional support” for PGx testing as a factor in our analysis and demonstrated that models including this factor have lower consistency and coverage than models without it, including our final selected model. We concede that having institutional support contributed to removing cost barriers in some prescriber settings but found that support alone was insufficient to drive PGx adoption.

### 4.2. Future Directions

Future studies aiming to characterize patient perspectives and preferences should take into consideration how different patient populations, such as Veterans, may have unique concerns and/or may be more likely than other patient populations to make certain health-related decisions [51].

Strengthening the evidence base and increasing awareness of the benefits of PGx testing may facilitate broader adoption among prescribers. Educational efforts that highlight both the potential cost savings associated with PGx testing and its ability to reduce the time required to identify optimal medications and dosages may further support clinical uptake. Future implementation efforts should also examine how clinical, organizational, and policy-level supports, including test coverage by health systems or translational research programs, as well as the culture and practices of genomics-enabled learning health systems (gLHSs), can be leveraged to promote adoption of PGx testing [52]. Additional factors to be evaluated might include how “medical necessity” is determined for the purpose of clinical implementation and eligibility for testing, and the role that clinical practice guidelines will play in terms of which tests are covered and for whom.

Comparing adoption in different contexts may offer further insights into trends we observed by investigating how organization-supported reimbursement practices, insurance coverage policies, and the availability of research funding to support PGx testing in a clinical setting can motivate more widespread adoption. The recent inclusion of pharmacogenetic testing as a Medi-Cal benefit in California provides an opportunity to examine whether, and to what extent, public insurance coverage policies influence clinical practice, reimbursement trends, and patient outcomes over time [50].

## 5. Conclusions

Our multi-site study evaluating prescribers of antidepressants is the first of its kind to use CNA to identify combinations of factors that may motivate clinical adoption of pharmacogenetic (PGx) testing in a variety of healthcare systems and clinical scenarios. We demonstrate that PGx tests were deemed by prescribers in the study to be beneficial and they are not concerned about cost as a barrier at the time of testing. The model with this combination of factors was best supported by both quantitative and qualitative data.

Our findings show that even when prescribers consider PGx testing to be potentially beneficial to their patients, contextual factors such as cost concerns are still a major barrier. This finding underscores the importance of healthcare and fiscal policies that promote equity in access to high-quality medical care, including precision medicine through genomic technology. Prescriber education about pharmacogenetics may be beneficial for enhancing the uptake of PGx testing in clinical practice, but there is also a need to bolster the evidence base demonstrating the utility of these tests so that prescribers understand the range of clinical scenarios and domains in which PGx testing can be beneficial to their patients.

Finally, creating health policies and healthcare systems with reimbursement strategies that support PGx testing can enhance the adoption of important public health genetics practice, so prescribers need no longer worry about the cost burden of a potentially beneficial clinical test. As Medicare reimbursement for PGx testing has become increasingly available under certain local coverage determinations when medical necessity and other requirements are met, ongoing educational efforts and EHR infrastructure could further support the implementation of PGx testing to prevent misperceptions about costs.

## Figures and Tables

**Table 1:** US Food and Drug Administration (FDA)-approved drug labels containing information about clinically actionable PGx interactions with antidepressants, consolidated from the ClinPGx table of drug label annotations (https://www.clinpgx.org/labelAnnotations; accessed on 11 June 2026). Additional regulatory agencies shown as sources of approved drug labels with actionable information about antidepressants include the European Medicines Agency (EMA), Health Canada/Santé Canada (HCSC), and Swissmedic.

Regulatory Agencies	Genes	Drugs
FDA	*CYP2C19*	citalopram
HCSC		
Swissmedic		
FDA	*CYP2D6*	dextromethorphan hydrobromide combined with bupropion hydrochloride
FDA	*CYP2D6*	fluoxetine
FDA	*CYP2D6*	fluoxetine
		olanzapine
FDA	*CYP2D6*	fluvoxamine
HCSC		
Swissmedic		
EMA	*CYP2D6*	vortioxetine
FDA		
Swissmedic		

Note: The FDA-approved labels included in this table contain clinically actionable pharmacogenomic information about gene–drug interactions that may inform prescribing decisions when genotype results are available. Inclusion in this table does not imply a recommendation for PGx testing.

**Table 2:** Characteristics of healthcare systems, clinical practice settings, level of institutional stage of PGx adoption, and individual/prescriber-level training in PGx testing, degree type, clinical area of specialty, and PGx testing adoption status.

**System-level (Institutional) Factors**					
**Study Site Id**	**1**	**2**	**3**	**4**	**Total**
**Distribution of Study Participants (N, % Total)**					
Patients Interviewed	5 (50%)	0 (0%)	0 (0%)	5 (50%)	10
Prescribers Included	4 (13.8%)	8 (27.6%)	5 (17.2%)	12 (41.4%)	29
**Healthcare Setting**					
*Regional system reaching rural areas*		X		X	2
*Urban academic medical center*	X		X		2
**NIH Clinical Genomics Network (Member or Affiliate)**					
*eMERGE*		X	X		2
*IGNITE*	X		X	X	3
**Program Stage (Institutional Level)**					
*Exploration*	X				1
*Preparation*		X			1
*Implementation (preemptive)*			X		1
*Expansion (preemptive and reactive)*				X	1
**Individual-level (Prescriber) Factors**					
**STUDY SITE ID**	**1**	**2**	**3**	**4**	**Total**
**Practice Setting (N, % Total)**					
*Inpatient*	1 (50%)	0 (0%)	1 (50%)	0 (0%)	2
*Outpatient*	3 (11.1%)	8 (29.7%)	4 (14.8%)	12 (44.4%)	27
**Prescriber Degree Type (N, % Total)**					
*MD*	4 (16.7%)	6 (25.0%)	5 (20.8%)	9 (37.5%)	24
*PA or NP*	0 (0%)	1 (25.0%)	0 (0%)	3 (75.0%)	4
*PharmD*	0 (0%)	1 (100%)	0 (0%)	0 (0%)	1
**PGx Exposure (N, % Total)**					
*Received Training* *(Med School/Residency/CME)*	2 (10%)	7 (35.0%)	3 (15.0%)	8 (40.0%)	20
*No Training Reported*	2 (22.2%)	1 (11.1%)	2 (22.2%)	4 (44.4%)	9
**PGx Adoption (N, % Total)**					
*Adopters*	2 (12.5%)	1 (6.25%)	3 (18.8%)	7 (43.8%)	16
*Non-Adopters*	2 (15.4%)	7 (53.9%)	2 (15.4%)	2 (15.4%)	13

**Table 3:** Factors with abbreviated titles as reported in the model outputs, with definitions for each factor and numerical coding scheme, as well as final calibrations for inclusion in the CNA matrix.

Factor (Abbreviation)	Description	Initial Coding	Final Calibration
Benefits	Beliefs about positive consequences and relative advantages of PGx	1 = negative effects 2 = mixed (positive and negative) 3 = only positive	0 = no benefits perceived (absence of positive) 1 = at least some perceived benefits (presence of positive beliefs)
Ability	Skills, procedural knowledge, or confidence about how to use pharmacogenetic testing in daily practice	Calibrated separately for each of 3 concepts as 1 = none 2 = moderate 3 = high	0 = low ability (did not have any skills, procedural knowledge, or confidence) 1 = some ability present (had any skills, procedural knowledge, or confidence)
Evidence strength and quality (Evidence)	Belief about the state of the evidence regarding use of pharmacogenetics in mental health	1 = negative (evidence favors not offering PGx testing) 2 = neutral 3 = positive (evidence is in favor of PGx testing)	0 = neutral or negative beliefs (evidence insufficient to support genetic testing for prescribing) 1 = positive beliefs about evidence
Cost barrier	Reported cost barrier at the time of PGx testing	1 = yes 2 = no	0 = neutral or cost was not reported as a barrier at the time of testing 1 = cost reported as a barrier at the time of testing
Institutional support (Support)	Presence of institutional or organizational support for PGx testing (e.g., financial subsidies, training, clinical PGx research)	1 = low 2 = moderate 3 = high	0 = no support 1 = at least some support
Acceptability (Accept)	Belief that pharmacogenetic testing is agreeable, palatable, or satisfactory	1 = disagree 2 = neutral 3 = agree	0 = disagree or neutral 1 = agree PGx is acceptable
Appropriateness (Approp)	Perceived fit of pharmacogenetic testing for setting or problem	1 = disagree 2 = neutral 3 = agree	0 = disagree or neutral 1 = agree (PGx is or may be appropriate)
Feasibility (Feas)	Belief that PGx testing can be successfully used or carried out within a given agency or setting	1 = disagree 2 = neutral 3 = agree	0 = disagree/neutral 1 = agree (pharmacogenetic testing is clearly feasible)

**Table 4:** CNA case matrix with fully calibrated data, which was used to run the final analysis. Cases (rows) are sorted by outcome, to facilitate visual comparisons of factor values for adopters (outcome = “1”) with those of non-adopters (outcome = “0”). For each factor, “1” = present; “0” = absent. Case matrix is sorted by outcome, so all adopter cases are highlighted in gray.

Outcome	Support	Ability	Cost Barrier	Benefits	Evidence	Appropriateness	Acceptability	Feasibility
0	1	0	1	0	0	0	0	1
0	0	0	1	1	1	1	1	0
0	1	1	1	0	0	0	0	1
0	1	0	1	1	1	1	0	0
0	0	0	1	1	0	1	1	1
0	0	0	1	1	0	1	1	0
0	0	1	1	0	0	0	0	1
0	0	0	1	1	1	1	1	1
0	0	0	0	0	1	0	0	1
0	1	0	1	0	0	0	0	1
0	1	1	0	1	0	0	0	1
0	1	1	0	0	0	0	0	1
0	1	1	1	1	1	1	1	1
1	0	1	0	1	1	1	1	1
1	1	0	0	1	0	1	1	0
1	0	0	0	1	1	1	1	1
1	1	1	0	1	1	1	1	1
1	1	1	0	1	1	1	1	1
1	1	1	0	1	1	1	1	1
1	1	0	0	1	0	1	1	1
1	1	1	0	1	1	1	1	1
1	1	1	0	1	1	1	1	1
1	1	1	0	1	1	1	1	1
1	1	1	0	1	1	1	1	1
1	1	1	0	1	1	1	1	1
1	1	1	0	1	1	1	1	1
1	1	0	0	1	1	1	0	1
1	1	1	0	1	0	1	0	1
1	1	1	0	1	1	1	1	1

**Table 5:** Top 10 most robust CNA models with performance metrics shown for each model. AA-Con and AA-Cov are the antecedent-adjusted consistency and coverage, respectively. C-Con and c-Cov are the contra-positive consistency and coverage, respectively. Fit-robustness score is normalized.

Model	Consistency AA-Con|c-Con		Coverage AA-Cov|c-Cov		Faithfulness	Complexity	Fit-Robustness Score
APPROP <-> OUTCOME	0.786	0.538	1.0	1.0	0.667	1	1.0
cost_barrier <-> OUTCOME	0.864	0.769	1.0	1.0	0.667	1	0.935
ACCEPT <-> OUTCOME	0.752	0.615	0.787	0.80	0.50	1	0.731
cost_barrier * EVIDENCE <-> OUTCOME	0.918	0.923	0.823	0.80	0.667	2	0.666
BENEFITS <-> OUTCOME	0.767	0.462	1.0	1.0	0.667	1	0.547
SUPPORT * ACCEPT <-> OUTCOME	0.907	0.923	0.787	0.750	0.571	2	0.50
cost_barrier * BENEFITS <-> OUTCOME	0.944	0.923	1.0	1.000	0.80	2	0.352
ABILITY * APPROP <-> OUTCOME	0.907	0.923	0.787	0.750	0.667	2	0.337
APPROP * FEAS <-> OUTCOME	0.845	0.769	0.902	0.909	0.60	2	0.337
SUPPORT * cost_barrier * EVIDENCE <-> OUTCOME	1.0	1.000	0.783	0.722	0.778	3	0.328

In CNA notation, * denotes AND, and <-> denotes a configurational relation meeting the minimal specified sufficiency and necessity fit criteria for the outcome. Uppercase factor names indicate the presence of the condition, and absence is denoted by lower-case letters. Because very few cases lacked feasibility, only the presence of feasibility was allowed to enter the model as a causal condition. Absence of feasibility was excluded as a candidate antecedent for adoption to reduce the risk that it would be used to describe a small pocket of cases rather than identify a credible causal condition.

## Data Availability

The raw data supporting the conclusions of this article include the transcripts and CNA matrix and will be made available by the authors on request.

## Abbreviations

The following abbreviations are used in this manuscript:

ADR	Adverse Drug Reaction
CNA	Coincidence Analysis
*CYP2C19*	Cytochrome P450, Subfamily IIC, Polypeptide 19
*CYP2D6*	Cytochrome P450, Subfamily IID, Polypeptide 6
EMA	European Medicines Agency
FDA	United States Food and Drug Administration
HCSC	Health Canada/Santé Canada
PGx	Pharmacogenetics
US	United States

## References

[1] GENDEP Investigators, MARS Investigators, STAR*D Investigators (2013). Common genetic variation and antidepressant efficacy in major depressive disorder: A meta-analysis of three genome-wide pharmacogenetic studies. Am. J. Psychiatry.

[2] Elmarasi M., Fuehrlein B. (2023). US Medicaid program: An analysis of the spending and utilization patterns for antidepressants from 2017 to 2021. Explor. Res. Clin. Soc. Pharm..

[3] Nagappan A., Miller A., Jain S., Oakes A.H. (2024). Stimulant, antidepressant, and opioid telehealth prescription trends between 2019 and 2022. JAMA Netw. Open.

[4] Sperber N.R., Roberts M.C., Gonzales S., Bendz L.M., Cragun D., Haga S.B., Wu R.R., Omeogu C., Kaufman B., Petry N.J. (2024). Pharmacogenetic testing in primary care could bolster depression treatment: A value proposition. Clin. Transl. Sci..

[5] Lemke A.A., Hulick P.J., Wake D.T., Wang C., Sereika A.W., Yu K.D., Glaser N.S., Dunnenberger H.M. (2018). Patient perspectives following pharmacogenomics results disclosure in an integrated health system. Pharmacogenomics.

[6] Allen J.D., Pittenger A.L., Bishop J.R. (2022). A scoping review of attitudes and experiences with pharmacogenomic testing among patients and the general public: Implications for patient counseling. J. Pers. Med..

[7] Virelli C.R., Ebrahimi M., Mohiuddin A.G., Tomasi J., Lisoway A.J., Herbert D., Marshe V.S., Kidd S.A., Ferenbok J., Kennedy J.L. (2023). User experiences of pharmacogenomic testing and opinions among psychiatry patients. J. Pers. Med..

[8] Cernat A., Samaan Z., Abelson J., Ramdyal A., Shaikh H., Vanstone M. (2024). Patient perspectives on pharmacogenomic (PGx) testing for antidepressant prescribing in primary care: A qualitative description study. J. Community Genet..

[9] Popejoy A.B. (2019). Diversity in precision medicine and pharmacogenetics: Methodological and conceptual considerations for broadening participation. Pharmgenomics Pers. Med..

[10] Loftus J., Levy H.P., Stevenson J.M. (2024). Documentation of results and medication prescribing after combinatorial psychiatric pharmacogenetic testing: A case for discrete results. Genet. Med..

[11] Zhou Y., Tremmel R., Schaeffeler E., Schwab M., Lauschke V.M. (2022). Challenges and opportunities associated with rare-variant pharmacogenomics. Trends Pharmacol. Sci..

[12] Swen J.J., van der Wouden C.H., Manson L.E., Abdullah-Koolmees H., Blagec K., Blagus T., Böhringer S., Cambon-Thomsen A., Cecchin E., Cheung K.C. (2023). A 12-gene pharmacogenetic panel to prevent adverse drug reactions: An open-label, multicentre, controlled, cluster-randomised crossover implementation study. Lancet.

[13] Skokou M., Karamperis K., Koufaki M.I., Tsermpini E.E., Pandi M.T., Siamoglou S., Ferentinos P., Bartsakoulia M., Katsila T., Mitropoulou C. (2024). Clinical implementation of preemptive pharmacogenomics in psychiatry. eBioMedicine.

[14] Bousman C.A., Arandjelovic K., Mancuso S.G., Eyre H.A., Dunlop B.W. (2019). Pharmacogenetic tests and depressive symptom remission: A meta-analysis of randomized controlled trials. Pharmacogenomics.

[15] Oslin D.W., Lynch K.G., Shih M.C., Ingram E.P., Wray L.O., Chapman S.R., Kranzler H.R., Gelernter J., Pyne J.M., Stone A. (2022). Effect of pharmacogenomic testing for drug-gene interactions on medication selection and remission of symptoms in major depressive disorder: The PRIME Care randomized clinical trial. JAMA.

[16] Chenchula S., Atal S., Uppugunduri C.R.S. (2024). A review of real-world evidence on preemptive pharmacogenomic testing for preventing adverse drug reactions: A reality for future health care. Pharmacogenomics J..

[17] Shriver S.P., Adams D., McKelvey B.A., McCune J.S., Miles D., Pratt V.M., Ashcraft K., McLeod H.L., Williams H., Fleury M.E. (2024). Overcoming barriers to discovery and implementation of equitable pharmacogenomic testing in oncology. J. Clin. Oncol..

[18] Xu L., Li L., Wang Q., Pan B., Zheng L., Lin Z. (2024). Effect of pharmacogenomic testing on the clinical treatment of patients with depressive disorder: A randomized clinical trial. J. Affect. Disord..

[19] Caudle K.E., Klein T.E., Hoffman J.M., Muller D.J., Whirl-Carrillo M., Gong L., McDonagh E.M., Sangkuhl K., Thorn C.F., Schwab M. (2014). Incorporation of pharmacogenomics into routine clinical practice: The Clinical Pharmacogenetics Implementation Consortium (CPIC) guideline development process. Curr. Drug Metab..

[20] Peruzzi E., Roncato R., De Mattia E., Bignucolo A., Swen J.J., Guchelaar H.J., Toffoli G., Cecchin E. (2025). Implementation of pre-emptive testing of a pharmacogenomic panel in clinical practice: Where do we stand?. Br. J. Clin. Pharmacol..

[21] Li D., Pain O., Fabbri C., Wong W.L.E., Lo C.W.H., Ripke S., Cattaneo A., Souery D., Dernovsek M.Z., Henigsberg N. (2024). Metabolic activity of CYP2C19 and CYP2D6 on antidepressant response from 13 clinical studies using genotype imputation: A meta-analysis. Transl. Psychiatry.

[22] Borden B.A., Galecki P., Wellmann R., Danahey K., Lee S.M., Patrick-Miller L., Sorrentino M.J., Nanda R., Koyner J.L., Polonsky T.S. (2019). Assessment of prescriber-perceived barriers to clinical use of pharmacogenomics during participation in an institutional implementation study. Pharmacogenetics Genom..

[23] van der Drift D., Simoons M., Koch B.C.P., Brufau G., Bindels P., Matic M., van Schaik R.H.N. (2023). Implementation of pharmacogenetics in first-line care: Evaluation of its use by general practitioners. Genes.

[24] Abdullah-Koolmees H., van Keulen A.M., Nijenhuis M., Deneer V.H.M. (2021). Pharmacogenetics guidelines: Overview and comparison of the DPWG, CPIC, CPNDS, and RNPGx guidelines. Front. Pharmacol..

[25] Sperber N.R., Cragun D., Roberts M.C., Bendz L.M., Ince P., Gonzales S., Haga S.B., Wu R.R., Petry N.J., Ramsey L. (2022). A mixed-methods protocol to identify best practices for implementing pharmacogenetic testing in clinical settings. J. Pers. Med..

[26] Ambuehl M., Baumgartner M., Epple R., Parkkinen V.P., Thiem A. (2025). cna: Causal Modeling with Coincidence Analysis.

[27] Whitaker R.G., Sperber N., Baumgartner M., Thiem A., Cragun D., Damschroder L., Miech E.J., Slade A., Birken S. (2020). Coincidence analysis: A new method for causal inference in implementation science. Implement. Sci..

[28] Atkins L., Francis J., Islam R., O’Connor D., Patey A., Ivers N., Foy R., Duncan E.M., Colquhoun H., Grimshaw J.M. (2017). A guide to using the Theoretical Domains Framework of behaviour change to investigate implementation problems. Implement. Sci..

[29] Damschroder L.J., Aron D.C., Keith R.E., Kirsh S.R., Alexander J.A., Lowery J.C. (2009). Fostering implementation of health services research findings into practice: A consolidated framework for advancing implementation science. Implement. Sci..

[30] Damschroder L.J., Reardon C.M., Widerquist M.A.O., Lowery J. (2022). The updated Consolidated Framework for Implementation Research based on user feedback. Implement. Sci..

[31] Proctor E., Silmere H., Raghavan R., Hovmand P., Aarons G., Bunger A., Griffey R., Hensley M. (2011). Outcomes for implementation research: Conceptual distinctions, measurement challenges, and research agenda. Adm. Policy Ment. Health.

[32] Weiner B.J., Lewis C.C., Stanick C., Powell B.J., Dorsey C.N., Clary A.S., Boynton M.H., Halko H. (2017). Psychometric assessment of three newly developed implementation outcome measures. Implement. Sci..

[33] Parkkinen V.P., Baumgartner M., Ambuehl M. (2025). frscore: Functions for Calculating Fit-Robustness of CNA-Solutions.

[34] Parkkinen V.P., Baumgartner M. (2023). Robustness and model selection in configurational causal modeling. Sociol. Methods Res..

[35] Hsieh H.-F., Shannon S.E. (2005). Three approaches to qualitative content analysis. Qual. Health Res..

[36] Caudle K.E., Hoffman J.M., Gammal R.S. (2023). Pharmacogenomics implementation: “A little less conversation, a little more action, please”. Pharmacogenomics.

[37] Whirl-Carrillo M., Huddart R., Gong L., Sangkuhl K., Thorn C.F., Whaley R., Klein T.E. (2021). An evidence-based framework for evaluating pharmacogenomics knowledge for personalized medicine. Clin. Pharmacol. Ther..

[38] Jessel C.D., Al Maruf A., Oomen A., Arnold P.D., Bousman C.A. (2022). Pharmacogenetic testing knowledge and attitudes among pediatric psychiatrists and pediatricians in Alberta, Canada. J. Can. Acad. Child Adolesc. Psychiatry.

[39] Apellaniz-Ruiz M., Barrachina J., Castro-Sanchez P., Comes-Raga A., García-González X., Gil-Rodriguez A., Lopez-Lopez E., Maroñas O., Morón R., Muriel J. (2024). Status of the implementation of pharmacogenetics in clinical practice in Spain: From regional to national initiatives. Drug Metab. Pers. Ther..

[40] Kaur G., Nwabufo C.K. (2024). Healthcare prescriber and patient perspectives on the implementation of pharmacogenetic-guided treatment in routine clinical practice. Pharmacogenetics Genom..

[41] Medwid S., Kim R.B. (2024). Implementation of pharmacogenomics: Where are we now?. Br. J. Clin. Pharmacol..

[42] Mosch R., van der Lee M., Guchelaar H.J., Swen J.J. (2025). Pharmacogenetic panel testing: A review of current practice and potential for clinical implementation. Annu. Rev. Pharmacol. Toxicol..

[43] Li L.J., Legeay S., Gagnon A.L., Frigon M.P., Tessier L., Tremblay K. (2024). Moving towards the implementation of pharmacogenetic testing in Quebec. Front. Genet..

[44] Wiss F.M., Jakober D., Lampert M.L., Allemann S.S. (2024). Overcoming barriers: Strategies for implementing pharmacist-led pharmacogenetic services in Swiss clinical practice. Genes.

[45] Roberts B., Cooper Z., Landery G., Stanley S., Majda B.T., Collins K.R.L., Akkari P.A., Hood S.D., Rodger J. (2025). Exploring perceived barriers and attitudes in young adults towards antidepressant pharmacotherapy, including the implementation of pharmacogenetic testing to optimize prescription practices. Front. Pharmacol..

[46] Cooper J., Pratt J., Park J., Fahim C., Lovnicki J.M., Groeneweg G.S.S., Carleton B., Straus S. (2024). Implementation of pharmacogenetic testing in pediatric oncology: Barriers and facilitators assessment at eight Canadian academic health centres. Pharmacogenomics J..

[47] Meli B.A., Fenech A.G., Cordina M., Ellul B., Agius E. (2025). Challenges in public policy for the implementation of pharmacogenetic tests in Europe. BioSocieties.

[48] Rim J.H., Kim Y.G., Kim S., Choi R., Lee J.S., Park S., Lee W., Song E.Y., Lee S.Y., Chun S. (2025). Clinical pharmacogenetic testing and application: 2024 updated guidelines by the Korean Society for Laboratory Medicine. Ann. Lab. Med..

[49] Manolio T.A., Rider R., Bult C.J., Chisholm R.L., Deverka P.A., Ginsburg G.S., Green E.D., Jarvik G.P., Mensah G.A., Narula J. (2025). Advancing the science of genomic learning healthcare systems. Learn. Health Syst..

[50] California Department of Health Care Services Policy Information for Biomarker and Pharmacogenomic Testing Coverage. https://mcweb.apps.prd.cammis.medi-cal.ca.gov/news/33051.

[51] Melendez K., Gutierrez-Meza D., Gavin K.L., Alagoz E., Sperber N., Wu R.R., Silva A., Pati B., Voora D., Hung A. (2023). Patient perspectives of barriers and facilitators for the uptake of pharmacogenomic testing in Veterans Affairs’ Pharmacogenomic Testing for the Veterans (PHASER) Program. J. Pers. Med..

[52] Pjevac M. (2024). The application of pharmacogenetic testing in psychiatry for treatment-resistant disorders: Optimal timing and implementation, a literature review. Eur. Psychiatry.

